# Management of recurrent pheochromocytoma in pregnancy in a young Ghanaian

**DOI:** 10.4314/gmj.v56i4.14

**Published:** 2022-12

**Authors:** Josephine Akpalu, Charlotte Ampong, Yacoba Atiase, Ernest Yorke, Charles Takyi, Jerry Coleman, Ebenezer O Darkwa, Nii A Adu-Aryee

**Affiliations:** 1 Endocrinology Unit, Department of Medicine and Therapeutics, University of Ghana Medical School; 2 Endocrinology Unit, Department of Medicine and Therapeutics, Korle Bu Teaching Hospital; 3 Department of Obstetrics and Gynaecology, Korle Bu Teaching Hospital; 4 Department of Anaesthesia, University of Ghana Medical School; 5 Department of Surgery, University of Ghana Medical School

**Keywords:** Pheochromocytoma, pregnancy, management, Ghana

## Abstract

The co-existence of pheochromocytoma and pregnancy is rare, with poor maternal and foetal outcomes. This is a case report of a young Ghanaian woman with a pre-existing diagnosis of recurrent pheochromocytoma who became pregnant and experienced elevated blood pressure in the third trimester with proteinuria and abnormal liver function. She was managed as an in-patient and delivered a live baby via caesarean section at 34 weeks after detecting intra-uterine growth restriction. Management of such cases by a multidisciplinary team is recommended for optimal outcomes.

## Introduction

Pheochromocytoma is a neuroendocrine tumour arising from the adrenal medulla.^1^ Pheochromocytomas secrete excessive amounts of one or more catecholamines responsible for the variable clinical features.^1^ The classic clinical manifestation of paroxysms of headaches, palpitations and excessive sweating associated with hypertension is seen in a minority of cases.^2^

Surgical resection is the treatment of choice for pheochromocytoma but does not lead to a cure in all cases.^1^,^3^ Recurrence rate of pheochromocytoma after surgery has recently been lower than previously documented.^4^ The co-existence of pheochromocytoma and pregnancy is rare, with dire maternal and foetal outcomes.^5^ In this report, we present the case of a young woman with a pre-existing diagnosis of recurrent pheochromocytoma who became pregnant and was managed by a multidisciplinary team in a tertiary health facility in Ghana amidst some constraints.

## Case Report

A 28-year-old female was referred to the surgical department of the Korle Bu Teaching Hospital with a 3-year history of intermittent right-sided abdominal pain and a retroperitoneal mass on abdominal computed tomography (CT) scan. She was subsequently referred to the endocrinology unit for evaluation for secondary hypertension.

Associated symptoms were palpitations, diaphoresis, heat intolerance and headaches. She had been diagnosed with hypertension and diabetes for three years and 2weeks, respectively. Physical examination revealed a blood pressure (BP) of 188/123mmHg and a right-sided firm mass upon gentle palpation of her abdomen. No signs suggestive of multiple endocrine neoplasia type 2 were elicited.

**Figure 1:**
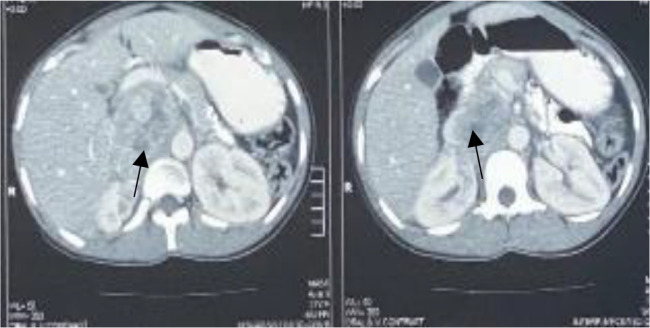
Abdominal CT scan; transverse section: showing a mass (7cm x 6cm) superior to the right kidney, with areas of necrosis (black arrows).

**Figure 2:**
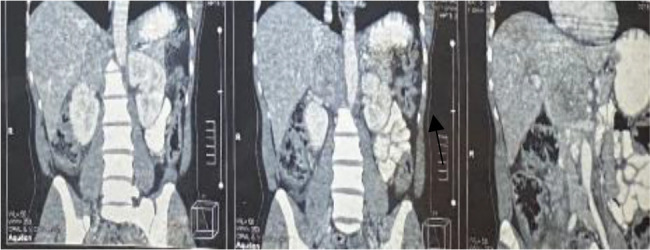
Abdominal CT scan; coronal section

A diagnosis of right adrenal pheochromocytoma was made based on the results of the 24-hour urinary normetanephrines (Table 1) and the findings of the abdominal CT scan. (Figures 1 and 2). Doxazosin was started for BP control, and bisoprolol was added because of persistent tachycardia.

**Table 1:** Pre- and Post-adrenalectomy biochemical results

Biochemical parameter	Pre-surgery	2 months post-surgery	4years post-surgery (Recurrence)	Normal range
**Potassium**	3.7mmol/L			3.5–5.0
**Estimated glomerular** **filtration rate**	>89umol/L			
**Fasting Plasma Glucose**	7.4mmol/L	5.2mmol/L		4.5–5.7
**Glycated haemoglobin**	6.3%	-	5.8%	4.8–5.7
**Urinary creatinine**	10.3mmol/24h	17.5	9.3	5.3 -15.9
**24-hour Normetanephrine** **excretion**	95,185nmol/day	22.7	42,587	606–2288
**Normetanephrine:Creatinine** **ratio**	9265.02umol/mol	109.21	4594.81	57–234
**24-hour Metanephrine** **excretion**	1138nmol/day	598	951	152–913
**Metanephrine:Creatinine** **ratio**	110.76umol/mol	34.21	102.59	17–91

She booked into ANC at 16weeks of gestational age. During review at 29weeks, the following were noted; BP of 160/100mmHg, proteinuria (trace), elevated liver enzymes and uric acid level (Table 2) with random plasma glucose of 5.6mmol/L.

**Table 2:** Antenatal clinic biochemical results

Biochemical parameter	Result	Normal ranges
**Aspartate aminotransferase**	71mmol/L	0–32
**Alkaline phosphatase**	160mmol/L	38–126
**Gamma glutamyl transferase**	60.9mmol/L	12–58
**Serum uric acid**	451umol/L	120–420

The histopathology report after a right adrenalectomy confirmed the diagnosis of pheochromocytoma. Post-operatively her BP, plasma glucose and urinary metanephrines normalized (Table 1) and all medications were stopped. She was lost to follow-up and presented 2years later after experiencing palpitations with elevated BP. A recurrent pheochromocytoma was suspected, doxazosin was restarted, and 24-hour urinary metanephrines were requested, but she returned with the results after 2years (Table 1). During that review, she complained of amenorrhea, and a urine pregnancy test was positive. She was referred to the obstetricians for antenatal care (ANC), and abdominal magnetic resonance imaging (MRI) was requested, which was not done due to financial challenges.

She was admitted and managed as hypertension due to recurrent pheochromocytoma with superimposed pre-eclampsia. The obstetrics and endocrinology teams co-managed her with the goals of controlling maternal BP, monitoring the foetus closely and delivering the fetus as close to term as possible. BP was controlled on Nifedipine and Doxazosin. Intravenous (IV) magnesium sulphate (4g slowly over 15 minutes) and intramuscular dexamethasone (6mg 12hourly for 48hours) were given for foetal neuroprotection and enhancement of lung maturation, respectively, in the event of imminent preterm delivery. Due to the risk of a pheochromocytoma crisis with steroid administration, she was closely monitored, and the endocrinology and intensive care units were alerted in case an emergency intervention was required.

Foetal surveillance measures included a daily foetal kick count and twice daily heart rate monitoring. The findings of obstetric ultrasound scans revealed a live singleton with an estimated foetal weight below the third percentile [indication of intrauterine growth restriction (IUGR)] and reduced end diastolic flow on doppler ultrasound. Based on these findings, an emergency caesarean delivery was performed at 34 weeks under a combined low dose spinal and epidural anaesthesia with invasive monitoring by the anaesthetist. This facilitated a more controlled onset of the anaesthetic block with minimal intraoperative hypotensive effects while providing adequate post-operative analgesia. Pre-operatively, IV magnesium sulphate and IV fluids were administered with perioperative fluid titration based on the patient's central venous pressure. IV glyceryltrinitrate was available to control intraoperative hypertension. Post-operatively mother remained stable under close monitoring in the intensive care unit (ICU) for 48 hours and was discharged on post-operative day five. A live singleton male baby weighing 1.7 kg was delivered with APGAR scores of 5 and 6 at one and five minutes, respectively. The baby was sent to the neonatal ICU due to prematurity and low APGAR scores. Baby was discharged after a month and is doing well having achieved all developmental milestones.

Postpartum her urine normetanephrine remained high and an abdominal CT scan showed a well-defined, homogenous mass adjacent to the right kidney (1.8x1.4x1.8cm) with no intralesional calcifications. The diagnosis of recurrent pheochromocytoma was confirmed, she was given genetic counselling and referred for a second adrenalectomy.

## Discussion

Recurrence rate of pheochromocytoma after surgery ranges between 6.5% and 16% with approximately 50% being malignant.^1^,^3^A recent metanalysis has however reported a lower recurrence rate of 3%.^4^ Factors associated with recurrence include familial syndromes due to genetic mutations, extra-adrenal tumors, young age at diagnosis, large tumour size (>5cm), and right-sided tumors.^6^,^7^ The latter three factors were present in our patient increasing the probability of tumour recurrence and emphasizing the importance of close longitudinal follow up.

Pheochromocytoma in pregnancy is rare (incidence of up to 1 in 54000 pregnancies) and is associated with adverse maternal and foetal outcomes.^8^ The clinical manifestations of pheochromocytoma in pregnant and non-pregnant individuals are similar are more prominent in the third trimester. This may be due to tumour stimulation by the growing uterus, uterine contractions, foetal movements and possible enhanced tumour progression by oestrogen.^5^,^9^,^10^ Our patient's BP control worsened in the third trimester in addition she had proteinuria and elevated liver enzymes which are suggestive of pre-eclampsia. Distinguishing pheochromocytoma from pre-eclampsia is essential but can be challenging.^5^,^11^ This distinction is vital to allow appropriate management and reduce the risk of poor maternal and foetal outcomes associated with delayed or missed diagnosis of pheochromocytoma.^5^,^12^

The large size of our patient's first tumour and its recurrence increases the likelihood of a familial syndrome or a malignant pheochromocytoma.1,7 The Pheochromocytoma of the Adrenal gland Scaled Score (PASS), a histo-pathological tool, is useful in identifying malignant tumours.^13^ Malignant pheochromocytoma during pregnancy are uncommon, however hereditary syndromes tend to be more prevalent.^12^–^14^ Commonly identified susceptible genes include *NF1, RET, VHL* and *SDH(A to D).*^16^ Offering genetic counselling and screening to young pregnant women with pheochromocytoma is therefore essential.^5^ In this case genetic testing was not performed because it is not readily available in our setting; however, genetic counselling was offered. Plasma and urine metanephrine levels remain normal during healthy pregnancy and are the recommended diagnostic biochemical test whereas MRI is the preferred radiological test.^16^

Management by a multidisciplinary team (endocrinologist, obstetrician, endocrine surgeon, anaesthetist, and geneticist) is recommended^10^ and should aim at adequate BP control to maintain uteroplacental circulation, optimal foetal and maternal surveillance for early identification of adverse events, appropriate timing of adrenalectomy, and selecting a suitable time and mode of delivery.^10^,^15^,^17^,^18^

Alpha-blockers phenoxybenzamine and doxazosin, for BP control, have been used with good outcomes.^14^,^16^ Beta-blockers can be used for a short period after alpha-blockade if BP is controlled and tachycardia persists. Large doses of beta-blockers have been associated with foetal bradycardia and IUGR, however, these rare adverse outcomes must be weighed against the benefits of treatment.^14^,^19^ Methyldopa, often used to manage hypertension in pregnancy, may worsen the symptoms of pheochromocytoma and should be avoided.^20^ Magnesium sulphate, which prevents the release of catecholamine from the tumour, blocks peripheral catecholamine receptors, and acts as a direct vasodilator, is useful in the management of pheochromocytoma.^15^

Adrenalectomy, the definitive management for pheochromocytoma, is recommended before 24weeks gestation. Beyond 24weeks, the enlarged uterus may restrict surgical access making medical therapy and adequate foetal monitoring while awaiting maturity before delivery the recommended approach.^16^, ^20^ Adrenalectomy can be performed after delivery. Surgery could not be performed for our patient during pregnancy since the MRI scan to locate the tumour was not done. Normal metanephrine levels 2-4weeks post-adrenalectomy suggest successful tumour resection; however, long-term bio-chemical follow-up is recommended to detect recurrence.^16^,^18^

Various factors including adequacy of medical treatment, gestational age, absence of foetal distress and patient's preference influence the delivery plan for pregnant women with pheochromocytoma.^15^ With adequate alpha blockade, sufficient analgesia and the avoidance of medications that can trigger crisis, vaginal deliveries with good outcomes have been documented.^13^,^22^ However caesarean section, regarded as a controlled procedure associated with a reduced risk of maternal mortality, tends to be the preferred mode of delivery.^23^

Elevated catecholamine levels can result in adverse maternal outcomes, including acute cardiac complications and stroke.5,24 The fetus, on the other hand, is shielded from the direct effects of high catecholamine levels due to degradation by placental catechol-O-methyltransferase and monoamine oxidase.^25^ However, uteroplacental vasoconstriction from maternal catecholamines and episodic hypertension can lead to poor foetal outcomes, including spontaneous abortion, growth restriction, premature delivery, and death.^24^,^25^ In our hospital, prematurity is the commonest cause of perinatal mortality and to improve perinatal outcome, our patient was given parenteral dexamethasone per the hospital's protocol. Despite the associated risk of precipitating a pheochromocytoma crisis with glucocorticoids, this was done. The necessary precautions were, however, taken to ensure a prompt response if the need arose.

The maternal and foetal mortality risk is approximately 50% in undiagnosed pheochromocytoma during pregnancy but reduces significantly if the diagnosis is made and appropriate treatment is given.^5^,^12^ Unrecognised pheochromocytoma during pregnancy, abdominal or pelvic tumour location and catecholamine levels of at least ten times the upper limit of the normal range have been associated with adverse maternal and foetal outcomes.^12^ Our patient's normetanephrine level was almost 20 times the upper limit of normal, exposing both mother and fetus to a high risk of adverse outcomes.

## Conclusion

Long-term surveillance of patients with pheochromocytoma following surgical intervention is crucial for the early detection and management of recurrence. Management of pheochromocytoma in pregnancy should be based on a patientcentred multidisciplinary approach to ensure optimal maternal and foetal outcomes.
